# Long-Term Surveillance of a Woodland Salamander Community with a Review of Long-Term Field Studies in Plethodontids

**DOI:** 10.3390/ani16030487

**Published:** 2026-02-04

**Authors:** Richard M. Lehtinen, Derek D. Calhoun, Jacob W. Gabriel, Hilary A. Edgington

**Affiliations:** Department of Biology, The College of Wooster, Wooster, OH 44691, USA; dcalhoun3366@gmail.com (D.D.C.); jwgabriel93@gmail.com (J.W.G.); hedgington@wooster.edu (H.A.E.)

**Keywords:** color morph, microhabitat use, hybridization, monitoring, phenology, surface activity, time series

## Abstract

Long-term ecological data are rare but are highly desirable for assessing responses to ongoing environmental change. To assess temporal trends in abundance over time and establish a baseline for future comparison, we monitored a plethodontid salamander community for ten years. From 2014 to 2023, we sampled forest plots at Wooster Memorial Park (OH, USA) using a regular and standardized monitoring scheme. Of nine salamander species detected, four were common enough to permit statistical analysis. Three species (*Eurycea bislineata*, *Plethodon cinereus* and *P. electromorphus*) had no statistically significant abundance trends over time. The slimy salamander (*P. glutinosus*), however, showed a statistically significant decline in abundance. We also report on ecological differences between *P. cinereus* and *P. electromorphus*, which occur in sympatry at this site. Specifically, we document significant microhabitat differences between these species, which are suggestive of competition avoidance. Additional data are presented on color morph frequency, body size, sexual dimorphism, frequency of hybridization, mate choices, and phenology of surface activity. As global environmental change accelerates, such baseline information is essential to track organismal responses. We also provide a brief review of other long-term field studies in plethodontid salamanders.

## Simple Summary

Long-term field studies of free-living animals are rare but are very useful for detecting changes over time. We monitored a natural salamander community for ten years (2014–2023) in a protected forest (Wooster Memorial Park, OH, USA) to assess changes and to provide baseline documentation of occurrence and abundance. Our results demonstrated that one species showed a significant decline in abundance over time, while three others showed no change. We also report on ecological differences between two closely related species that occur together at our study site, as well as a variety of other natural history information from these species. Finally, we provide a brief review of other long-term field studies in plethodontid salamanders.

## 1. Introduction

By many estimations, we are currently in the 6th mass-extinction event in the history of life on Earth. As impacts from a growing human population accelerate, biotic declines are seemingly everywhere [1,2]. Amphibians are among the hardest hit in this biodiversity crisis with significant negative impacts from climate change, habitat loss, and disease [3,4,5].

To track and understand the consequences of these biotic losses and to target interventions to at-risk species, long-term data on trends in distribution and abundance are urgently needed [6,7,8]. Such long-term monitoring data is acquired by regularly repeated data collection efforts using appropriate and standardized methodologies. And yet, even with long-term monitoring data, it can be quite challenging to detect declines in species abundance before they become obvious, especially in rare or difficult-to-detect species [9,10]. The demographic declines in births or increases in deaths (or both) that cause population sizes to fall often take intensive study to document and may take decades, centuries or millennia to play out [11], and limited datasets can have insufficient statistical power to detect declines [6].

Even in the absence of declines, amphibian populations are frequently characterized by strong natural variation in recruitment, making the detection of population declines an even greater challenge [12,13]. Further, population monitoring programs are frequently short-term and are often not started until declines are suspected, such that pre-decline baseline abundance data are often not available or are of dubious quality [14,15]. Worse yet, population monitoring and related “natural history studies” are often perceived as low-quality science among scientists and are therefore not often encouraged [16,17,18]. But to be able to respond to declines with management or conservation initiatives presupposes that we know the declines exist. Long-term population monitoring studies are thus critical for such biodiversity conservation initiatives [6,8,15,19,20].

In addition to detecting declines, long-term monitoring studies are also useful for several other important reasons. Often, organismal field studies might only last three or fewer years, but whether those years are representative or exceptional is usually unknown. A longer temporal data sequence is especially useful when examining slow or highly variable phenomena, when studying long-lived organisms, or when examining the role of rare or episodic events [16]. Second, a longer temporal sequence has the additional benefit of accumulating large sample sizes. These larger samples can enable the statistical detection of more subtle patterns in the data than usually would be possible. For example, even uncommonly encountered species, infrequent environmental changes or rare behaviors become capable of scientific scrutiny when enough instances have been observed. A long-term approach is especially useful in such a group as plethodontid salamanders, which are widely known for their subterranean activity patterns that strongly respond to changing environmental conditions at the surface [21,22,23].

Much of salamander diversity is concentrated in the family Plethodontidae, which makes up well over half of all salamander species globally (519 out of 825 currently known species; [24]). Plethodontid salamanders are not only taxonomically diverse but also ecologically important. These creatures are both important predators and important prey in their ecosystems, contribute significantly to soil dynamics, and are significant indicators of ecosystem integrity [25,26,27]. Recent estimates of abundance for just one terrestrial species (*Plethodon cinereus*, the eastern red-backed salamander) put median density at nearly 10,000 individuals per hectare [28], indicating a numerically important role of plethodontid salamanders in food webs.

Due to its large geographic range, local abundance and tractability in both the field and laboratory environments, *P. cinereus* has become an important model system in ecology, evolution and behavioral research [29,30]. While many aspects of its biology have been well-studied, two are particularly relevant here. The first is the presence of color polymorphism in this species. While numerous color morphs of *P. cinereus* exist [31], the striped and unstriped color morphs are the most common and have received the most attention. Many studies have reported an association between the frequency of these color morphs and the climatic conditions at a site: warmer and drier climates are associated with an elevated proportion of the unstriped morph, and cooler and wetter climates are associated with an elevated proportion of the striped morph [31,32,33,34]. The color morphs differ in numerous other ways as well, including in physiology [35], diet [36], predation risk [37], disease infection [38], mate choice and territorial behavior [39]. From an evolutionary perspective, polymorphic species represent useful study subjects. Since both color morphs occur within the same environment, they allow the possibility of identifying differential selection between them [40]. Identifying potential population divergence in sympatry is a key component of recent thinking on ecological speciation [40,41].

The second important aspect of *P. cinereus* biology that is relevant here is its interactions with congeners. Given its great abundance and large geographic range, *P. cinereus* interacts with numerous other small-bodied *Plethodon*. As interspecific competition is often strongest between closely related species, this can be an important selective force influencing evolutionary trajectories [42]. Previous work has shown evidence for divergence in behavior, body size, habitat use, head morphology, and diet when in sympatry with other small *Plethodon* species such as *P. hoffmani*, *P. hubrichti* and *P. shenandoah* [43,44,45,46,47]. Observations of interactions between *P. cinereus* and congeners can provide insight into the consequences of secondary contact, as is the case with *P. electromorphus* (the northern ravine salamander), another small *Plethodon* that has areas of sympatry with *P. cinereus*. In staged encounters in the laboratory, Deitloff et al. [48,49] showed that *P. cinereus* individuals tended to be more aggressive than *P. electromorphus* individuals. Individuals of both species from sympatric locations also showed heightened aggression compared to those from allopatric locations, suggesting selection for elevated aggression in sympatry [49]. Given these results and since these two species co-occur less commonly than expected by chance [50,51], competitive exclusion has been hypothesized as a possible outcome of their interactions [49]. If competitive interactions between *P. cinereus* and *P. electromorphus* are asymmetric, then declines in *P. electromorphus* abundance over time might be expected in sympatry [51].

Another possibility is that in sympatry, one or both species may have evolved traits to facilitate local coexistence since there should be selection for any traits that reduce interspecific competition [52,53]. While Deitloff et al. [54] found no evidence for differences in diet, cover object use or cranial morphology between allopatric and sympatric populations of these species, Hedeen [55] reported that *P. electromorphus* used drier microhabitats than *P. cinereus*, suggesting that microhabitat specialization may help limit niche overlap in sympatry. Waldron et al. [56] did not confirm this pattern and generally found broad overlap in conditions used by these two species. Thus, the available data are unclear on whether populations of these two species are stable in sympatry and what mechanisms might be preventing competitive exclusion.

While competition is an important aspect of the interaction of *P. cinereus* with congeners, some evidence of hybridization has been uncovered as well. Carpenter et al. [57] found limited evidence of hybridization between *P. cinereus* and *P. shenandoah* (but see [58]), and Bayer et al. [59] found no evidence for hybridization between *P. cinereus* and either *P. sherando* or *P. serratus*. On the other hand, Highton [50] identified hybridization between *P. cinereus* and *P. electromorphus* in northeastern Ohio using allozymes. Lehtinen et al. [60] later confirmed this result using single-nucleotide polymorphism data. A re-analysis of the Lehtinen et al. [60] data by Kuchta et al. [61] estimated that at Wooster Memorial Park (the most densely sampled site, hereafter WMP), between 8 and 33% of genotyped individuals were hybrids, depending on the analysis used. Thus, it appears that interspecific gene flow may be somewhat common at this particular site. Unfortunately, due to the challenge of identifying hybrids in the field [62], most studies of hybridization in plethodontids are focused on genotyping [63], limiting our understanding of these interactions. For example, how extensively hybrids and parentals overlap ecologically in sympatry or how the frequency of hybrids might differ in different microhabitats or in different environmental contexts is often poorly known.

Here, we report on a 10-year monitoring study of the plethodontid salamander community at a single study site. While our motivation was initially to provide baseline abundance data in the context of amphibian declines, the long-term nature of this project also facilitated the examination of a variety of additional topics, as the accumulated samples over many years permitted statistical examination of topics otherwise difficult to assess. Specifically, our goals were to

(1) Provide baseline salamander abundance and distribution information for future comparisons and to assess temporal trends in abundance over time using a standardized monitoring scheme.
(2) Assess factors affecting salamander abundance and surface activity patterns.
(3) Examine ecological differences between *P. cinereus* and *P. electromorphus* (and their hybrids) in sympatry.
(4) Examine ecological differences between the striped and unstriped color morphs of *P. cinereus*.
(5) Provide a variety of additional natural history information on *P. cinereus* and *P. electromorphus* at WMP, including: body size and sexual dimorphism, frequency of hybridization, mate choice, phenology, and frequency of tail damage.
(6) Review and synthesize other long-term field studies on plethodontid salamanders.

## 2. Methods

### 2.1. Study Site

Wooster Memorial Park is a ~172-hectare protected area located near the city of Wooster, OH, USA. Habitats include old growth, mature second growth and young deciduous forest, old agricultural fields, and riparian habitats associated with a second-order stream and its tributaries. The larger landscape is primarily agricultural with scattered small urban areas.

### 2.2. Field Sampling

**Figure 1:**
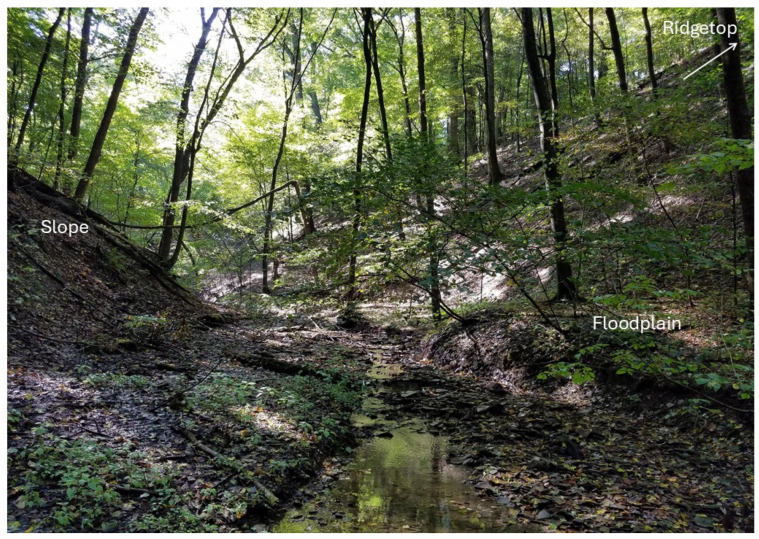
Photo of representative floodplain, and slope forest microhabitats adjacent to a first-order stream at Wooster Memorial Park, OH, USA. The ridgetop forest microhabitat would be found further upslope on the hilltop, as indicated by the arrow. Photo by R.M.L.

Starting in the fall of 2010, 10 × 25 m plots were sampled in three forested microhabitats at WMP. These plots were sampled specifically to examine potential microhabitat differences between *P. cinereus*, *P. electromorphus* and their hybrids. The three microhabitats were floodplain forest, ridgetop forest and the slopes lying between them (Figure 1). Floodplain forests were defined as bottomland forests found in the vicinity of creeks or rivers with little topography. Ridgetop forests were defined as upland forests not directly adjacent to creeks or rivers that also had little topography. The slope microhabitat was defined as hillside forests that occurred with substantial topographic relief, typically between 20 and 40 degrees.

Once a location at WMP was identified that met the microhabitat characteristics defined above, a frisbee was thrown haphazardly, and its landing point became one corner of a plot. Each 10 × 25 m plot was subsequently searched a single time for 30 person-minutes during daylight hours. The number of researchers searching the plot varied from one to seven and primarily consisted of undergraduates at the College of Wooster on biology class field trips, but also occasionally included citizen science volunteers. Researchers manually turned over rocks, logs, bark and other debris on the forest floor and sifted through leaf litter searching for salamanders until the end of the sampling period. Salamanders found were hand-captured using sterile gloves and placed in an unused plastic bag.

After sampling was complete, all salamanders were field identified by R.M.L., with the exception of a small number reported in [64,65] that were identified by those authors after extensive identification training. These counts of surface-active salamanders were used as an indicator of relative abundance since time and resource limitations precluded a mark-recapture study. For *P. cinereus*, *P. electromorphus* and probable *P. cinereus* × *P. electromorphus* hybrids, we also collected information on the cover object type (rock, log or leaf litter), the soil temperature (taken in shade with a Nubee NUB8500H infrared thermometer, Shenzhen Jumaoyuan Science and Technology Co., Ltd. Shenzhen, China), soil moisture (taken at a depth of ~5 cm with a soil moisture meter, model DSMM500, General Tools, Secaucus, NJ, USA) and leaf litter depth (taken with a ruler, averaged from four measurements) at each capture point. These data were collected sporadically in 2010 and regularly starting in 2011.

From the beginning of the study, we were aware of previous reports of hybridization between *P. cinereus* and *P. electromorphus* in our geographic area (first determined via protein gel electrophoresis by [50] and later confirmed with DNA sequence data by [60,61]). Therefore, individuals of these species were examined carefully for morphological intermediacy in the field using the morphological and coloration criteria reported in [60]. In the analyses that follow, individuals that were identified as probable hybrids in the field are categorized separately from individuals that were identified as pure *P. cinereus* or pure *P. electromorphus*. It is important to emphasize, however, that none of the probable hybrids reported on here were genotyped. While in earlier studies, many individuals field-identified as hybrids were later confirmed as hybrids with molecular data [60,61], data and analyses of probable hybrid individuals should, nonetheless, be interpreted cautiously.

Following the collection of ecological data, salamanders were then immediately released at their capture point. A total of 72 plots were sampled in this fashion in the years 2010, 2011, 2014 and 2015. Sampling was conducted in April, May, September and October, and plots were only sampled once.

### 2.3. Long-Term Monitoring Plots

In May of 2014, twelve permanent plots (10 × 10 m) were established at WMP for the purposes of long-term population monitoring of the entire salamander community. Four plots were placed in floodplain forests, four plots were placed in ridgetop forests and four plots were placed on north-facing slopes (as defined above in Figure 1; GPS coordinates of plot locations are in Table 1). There was little variation in canopy cover among plots, as all were in a mature forest at the same locality. Each plot was a minimum of 130 m from any other plot (average distance 576 m). To minimize the potential influence of edge effects, all plots were also placed more than 25 m from a habitat edge. Sampling began in May 2014 and continued until November 2023, with the goal of sampling each plot twice each year. A total of 230 plot sampling events occurred during the study period, with each plot being visited between 18 and 20 occasions over the ten-year period (usually two times per year). The first plot sampled each year was randomly determined. From one to four plot sampling events occurred per sampling day, rotating among microhabitat types.

Sampling occurred using the same methods described above and all salamanders were identified by R.M.L. Since re-visiting plots could conceivably impact salamander abundance due to the inevitable disturbance associated with manual sampling, a minimum of 30 days typically elapsed between additional sampling of the same plot in the same year (mean number of days elapsed between sampling events: 128 (range: 15–209). Due to the long-term nature of this study, care was also taken to minimize disturbance within the plots while sampling. Unlike the initial sampling described above, we sampled the long-term monitoring plots in all months between April and November to be able to detect species with various phenological patterns. During the sampling of the long-term monitoring plots for salamanders, we also counted all earthworms encountered, since earthworms are non-native to this portion of Ohio and other studies have suggested they have important interactions with salamanders (e.g., [66]). We did not use mustard-based sampling methods for earthworms [67] since this would have created unacceptable habitat modification in a long-term monitoring study. All earthworms at WMP that we have identified are in the genus *Lumbricus*.

**Figure 2:**
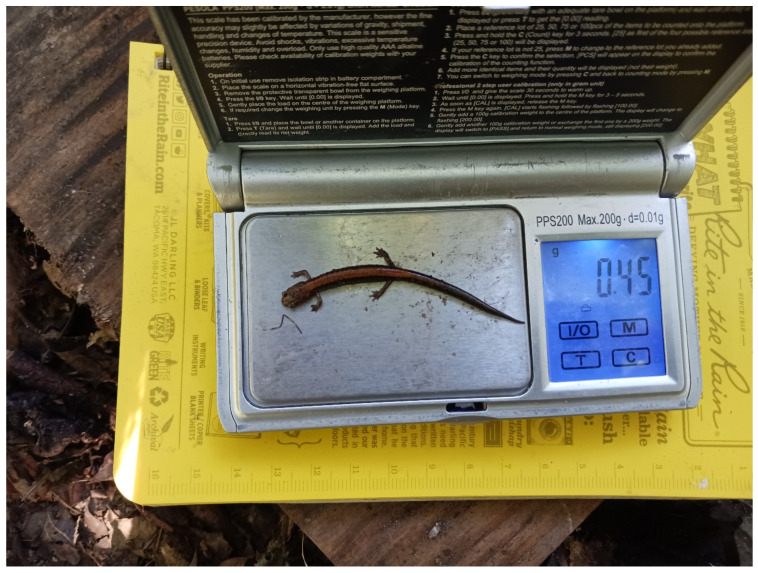
*Plethodon cinereus* individual on a digital balance with the metric scale in the image. Photographs such as these were used to determine body-size measurements (see text for details). Photo by R.M.L.

For *P. cinereus*, *P. electromorphus* and probable *P. cinereus* × *P. electromorphus* hybrids, we also collected data on body size, life stage, sex, body mass and reproductive status. In the field, each captured individual was weighed on a calibrated portable field scale (Pesola model PPS200, manufactured by Pesola AG, Schindellegi, Switzerland) and photographed dorsally with a metric ruler in the image using a Samsung Galaxy smartphone camera (model SM-A136U1 Samsung Electronics, Suwon, South Korea; see Figure 2). Since sexing plethodontid salamanders can sometimes be challenging, all captured individuals were visually inspected in the field for signs of a mental gland, cirri, a swollen cloaca or naso-labial groove [68]. For purposes of analysis, all individuals less than 22 mm SVL were classified as hatchlings; all individuals larger than 32 mm SVL were classified as adults, and individuals between 22 and 32 mm SVL were classified as juveniles, based on data from *P. cinereus* [69]. Occasionally, eggs were visible through the body wall, and these individuals were classified as gravid females. Subsequently, photographs were opened in the ImageJ software package version 1.53e [70] and, after calibration to the metric ruler in the photograph, the line tool was used to estimate snout–vent length (SVL) and total length (TL). Total length was measured from the tip of the snout to the tip of the tail, drawing as many lines as necessary to complete this measurement. This method is consistent, efficient and reduces animal handling times (see [71] for more details on photographic size measurements). However, since the vent is not visible from a dorsal photograph, we instead measured to the midpoint between the hindlimbs as a reliable landmark to estimate SVL. Due to this methodological difference compared to manual approaches (e.g., hand measuring with calipers), our SVL measurements may not be directly comparable to other studies but are nonetheless internally consistent. Our estimates of TL should be comparable to those in the literature. We also noted tail damage when apparent. On occasion, we found mated pairs under cover objects. These were defined as an adult male and an adult female found within 30 cm of each other under the same cover object during the breeding season, following [39]. After completing the sampling for each plot, we took five soil surface temperature measurements, five soil moisture measurements and five leaf litter depth measurements at five haphazardly selected locations in each plot.

### 2.4. Literature Review Methods

To summarize available information on published long-term field studies of plethodontid salamanders, we conducted a literature review using Google Scholar and Web of Science with the following keywords: plethodont, monitor, time series, baseline, decline and temporal trend. We identified additional relevant papers from the literature cited in papers found from our database searches and from Table 1 in [26]. Additional studies that fit our criteria (below) but did not come up in our literature search were suggested for inclusion by colleagues. To include in our list of long-term field studies, we only considered field studies conducted for a minimum of five consecutive (or near-consecutive) years at a single study site. Or, if multiple study sites were used, we still required at least one of the study sites to have a minimum of five consecutive years of field data. This excludes some collection-based studies with different goals that tend to maximize spatial rather than temporal coverage (e.g., [72,73]). Here, we focus on studies that sampled for salamanders regularly and repeatedly with known sampling effort rather than more haphazard, irregular sampling schemes. While there is no universally accepted definition of what constitutes a long-term ecological study, we chose a minimum of five years duration since this likely encompasses at least one generation of most species of plethodontid salamanders [74,75]. Re-analyses of the same dataset were noted but not counted as a separate study.

### 2.5. Statistical Analysis

#### 2.5.1. Analyses from Long-Term Monitoring Plots (2014–2023)

We examined the environmental variables of leaf litter depth, soil temperature, and soil moisture using a Mann–Kendall test for monotonic trend to ensure an environmental trend was not influencing salamander abundance trends. Only four species (plus the hybrid form) were detected sufficiently often to permit statistical testing. These included the following: *Eurycea bislineata*, *Plethodon cinereus*, *P. electromorphus* and *P. glutinosus*. Mann–Kendall tests were used to examine trends in abundance in these species over time using pooled plot means for each species in each year. We also examined the stationarity of each species’ abundance data using an Augmented Dickey–Fuller test. To examine the factors influencing abundance and surface activity patterns (i.e., counts), we used generalized linear mixed models (GLMMs) using a negative binomial distribution and log link function. We used the negative binomial to address potential overdispersion and because they outperformed models using the Poisson distribution (as measured by AIC, see also [76]).

We first conducted GLMMs for each species to determine the variables influencing surface activity (and therefore, detection). In these analyses, we used survey-specific soil temperature, soil moisture, leaf litter depth, days since a soaking rain event and day of year (Julian day) as fixed effects and plot identity as a random effect. Since Augmented Dickey–Fuller tests indicated non-stationarity for all species (see Results), we also added year as a fixed effect to account for some of the temporal variation. Days since soaking rain was defined as the number of days since a ≥5 mm rain event (following [77]). Rainfall estimates were determined from wunderground.com (accessed 27 May 2025) from the nearest weather monitoring station, located 4.8 km from the study site.

Subsequently, we conducted another GLMM examining abundance patterns for each species using microhabitat type and maximum earthworm abundance as fixed effects. Maximum earthworm abundance per plot per year was used instead of the number quantified on each survey, since earthworms also vary in detectability, and the maximum number is more likely to be representative of the true earthworm abundance in each plot. Variables found to be significantly associated with activity in the first round of GLMMs were included in these models as random effects to help control for the effects of detection probability on our abundance models. Year and plot identity were also included as a random effect for each model.

To assess the potential influence of repeated plot sampling on subsequent salamander counts, we conducted two analyses. First, we calculated the number of days that elapsed between the first and second visit of each plot in each year and compared it with the number of salamanders detected in the second visit using a Spearman correlation analysis. If sampling disturbance during the first visit negatively influenced detection on the second visit, there should be a significant positive correlation between the number of days elapsed between the first visit and the number of salamanders detected on the second visit. Our second analysis to examine the influence of repeated plot sampling on salamander detection calculated the difference in the number of salamanders detected on the first visit and the second visit of each plot in each year. If the second visit had consistently fewer salamanders detected compared to the first visit, the mean difference should be negative and significantly different from zero. We tested this possibility using a one-sample t-test.

Lastly, to empirically estimate detection probabilities for each species, we used an occupancy modeling approach [78]. After assembling detection histories for each survey of each plot in each year, we constructed a multi-season occupancy model separately for each species using the default parametrization in the software package Presence version 2.15.18 [79].

#### 2.5.2. Analyses from All Plots (2010–2023, *P. cinereus* and *P. electromorphus* Only)

To analyze ecological differences between *P. cinereus*, *P. electromorphus* and probable hybrids, we used a multivariate general linear model to test for differences in continuous variables like soil temperature, soil moisture and leaf litter depth, after confirming test assumptions were met. If assumptions were not met, non-parametric alternatives were used with Bonferroni correction for multiple comparisons. A similar approach was used to compare environmental conditions at capture locations between the striped and unstriped morphs of *P. cinereus* and to compare body size differences both between species and among sexes within species.

For categorical data like microhabitat type and cover object type, we used X^2^ goodness-of-fit tests to assess preferences and X^2^ contingency tests to assess differences in preferences between species. To calculate an index of relative body condition for *P. cinereus*, *P. electromorphus* and probable hybrids, we used a linear regression of body mass (dependent variable) versus SVL (independent variable) for all individuals (after confirming linearity and the absence of outliers). After confirming the normality and homoscedasticity of the residuals, we used the unstandardized residual as a measure of body condition [80]. A general linear model was used to compare body condition among *P. cinereus*, *P. electromorphus* and probable hybrids. Due to variation in missing data over time, sample sizes often differ slightly among different analyses. Statistical analyses were performed either in SPSS version 29.0.1 or in R version 4.1.5. Graphs were also made using R version 4.1.5.

## 3. Results

### 3.1. Long-Term Monitoring Plots (2014–2023)

**Figure 3:**
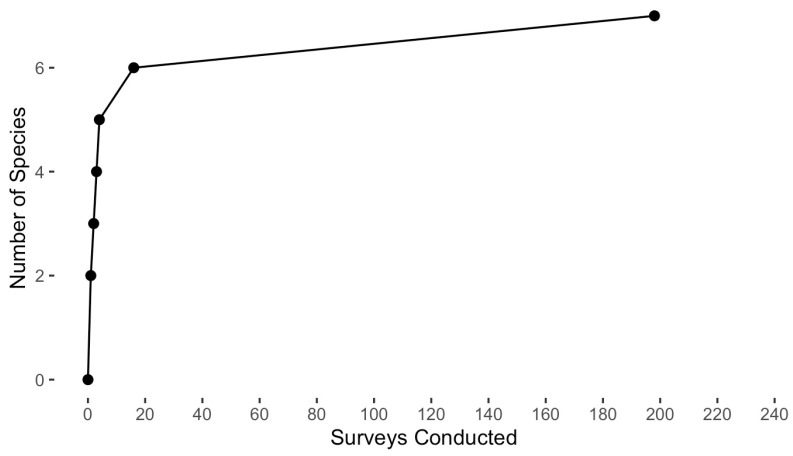
Species accumulation curve. A total of 7 plethodontid salamander species were detected during formal plot searches at WMP (n = 230 searches of twelve permanent forest plots, 2014–2023).

A total of nine species of salamanders were detected during this study (plus one hybrid form), representing a total of 679 individuals (Table 2). Five frog species and three snake species were also detected (Table 2). Common species were detected early on in the study, but some rare species were not detected until late in the study (Figure 3). For statistical power reasons, we restrict our analyses below to the four most commonly encountered species (*E. bislineata*, *P. cinereus*, *P. electromorphus* and *P. glutinosus*).

### 3.2. Temporal Trends

**Figure 4:**
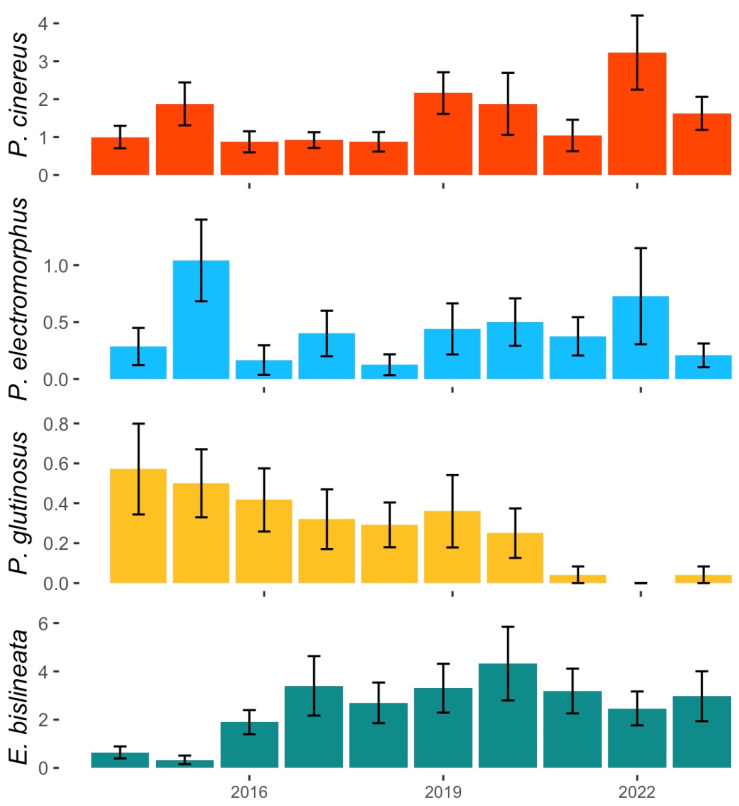
Abundance trends for *P. cinereus*, *P. electromorphus*, *P. glutinosus* and *E. bislineata* from the long-term monitoring plots at Wooster Memorial Park (2014–2023). For each year, the mean number of individuals encountered (all plots, all sampling events pooled) is shown ± SE. Linear regressions of pooled mean abundance versus time were non-significant for all species except *P. glutinosus* (T = −0.854, *p* < 0.001).

We observed no significant change over time in leaf litter depth (T = −0.0667, *p* = 0.85803), soil moisture (T = 0.0667, *p* = 0.85803), or soil temperature (T = 0.0222, *p* = 1). The annual mean number of individuals detected in plot searches (all plots pooled) had no significant relationship with time for *P. cinereus* (T = 0.296, *p* = 0.293), for *P. electromorphus* (T = 0.0667, *p* = 0.858), or for *E. bislineata* (T = −0.156, *p* = 0.592; Figure 4). However, there was a significant decrease over time detected for *P. glutinosus* (T = −0.854, *p* < 0.001; Figure 4). We could not reject the null hypothesis that abundance data were non-stationary for *P. cinereus* (DF = −2.147, *p* = 0.516), *P. electromorphus* (DF = −2.586, *p* = 0.349), *P. glutinosus* (DF = −1.407, *p* = 0.798), and *E. bislineata* (DF = −2.267, *p* = 0.471). Baseline density estimates from plot searches for each species can be found in Table 3.

### 3.3. Factors Influencing Surface Activity

**Figure 5:**
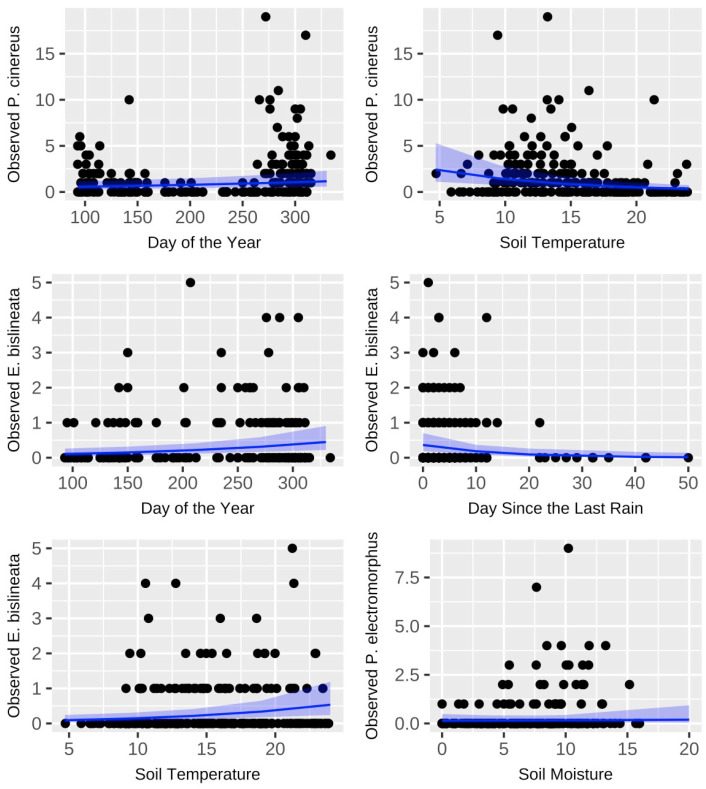
The effects ± 95% CI of each variable found to be significantly associated with salamander activity for each species examined. A generalized linear mixed model with negative binomial structure was used to determine whether there was a significant relationship between activity levels and the fixed effects: soil temperature, soil moisture, leaf litter depth, days since a soaking rain event and day of year. Black dots indicate counts for individual plot surveys. Blue shading indicates the 95% confidence interval for each depicted relationship. Statistical results can be found in Table 4. Models were visualized using the “effects” and “ggplot2” packages in R.

Influences on surface activity varied substantially among species. For example, surface activity in *P. cinereus* was significantly influenced by both time of year and soil temperature, while in *P. electromorphus*, only soil moisture was important (Table 4; Figure 5). Surface activity in *E. bislineata* showed some similarities to *P. cinereus*, as they had similar responses to time of year. Days since a soaking rain event were also influential (Table 4; Figure 5). Increasing soil temperature, however, had a significant positive relationship to surface activity in *P. cinereus* but a significant negative relationship in *E. bislineata* (Table 4; Figure 5). We identified no statistically significant variables influencing surface activity for *P. glutinosus*. There was no significant correlation between the number of days elapsed between plot searches and salamander abundance on the second visit (rho = 0.110, *p* = 0.259, n = 108). The mean difference between salamander abundance on the first visit and salamander abundance on the second visit to each plot was not significantly different from zero (t = 1.42, df = 107, *p* = 0.16, mean difference 0.56). Detection probabilities calculated using an occupancy modeling approach are as follows: *P. cinereus* (0.602, SE = 0.045), *P. electromorphus* (0.448, SE = 0.075), *P. glutinosus* (0.361, SE = 0.056), *E. bislineata* (0.367, SE = 0.039).

### 3.4. Factors Influencing Abundance

The abundance of all four species analyzed (*P. cinereus*, *P. electromorphus*, *P. glutinosus* and *E. bislineata*) was strongly influenced by forest microhabitat type (Table 4). The slope microhabitat maximized abundance for *P. cinereus*, *P. glutinosus* and *E. bislineata*. In contrast, *P. electromorphus* was most abundant in floodplain microhabitats (Table 3 and Table 4). For all species, abundance was lowest in the ridgetop microhabitat. The maximum abundance of earthworms was a significant negative predictor of abundance in *P. electromorphus*, while in *P. cinereus*, *P. glutinosus* and *E. bislineata*, there was no significant relationship detected (Table 4).

### 3.5. Seasonal Activity Patterns

**Figure 6:**
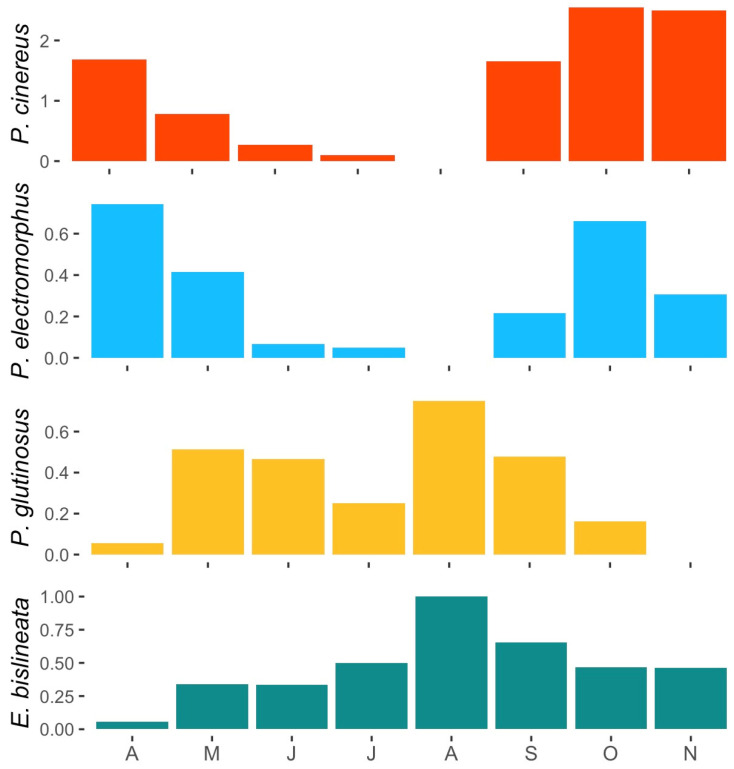
Number of individuals encountered per survey conducted (April–November) for *P. cinereus*, *P. electromorphus*, *P. glutinosus* and *E. bislineata* from the long-term monitoring plots at Wooster Memorial Park (2014–2023).

Of the four commonly encountered species in this study (*E. bislineata*, *P. cinereus*, *P. electromorphus* and *P. glutinosus*), several different patterns of annual activity were identified (Figure 6). *P. cinereus* and *P. electromorphus* had similar patterns with peaks in April and May, periods with few detections in June, July and August, and another peak in September, October and November. *P. glutinosus*, on the other hand, had many detections from May through September (with a peak in August) and relatively few in April or November. *E. bislineata* generally had relatively few detections early in the year (April–June) but substantially more later (July–November). Just examining hatchling occurrence for *P. cinereus* and *P. electromorphus*, the appearance of *P. cinereus* hatchlings is strongly concentrated in October and November (32/41, 78% of all hatchling detections). In contrast, the appearance of *P. electromorphus* hatchlings is strongly concentrated in April and May (13/20, 65% of all hatchling detections).

Ecological differences between *P. cinereus* and *P. electromorphus* (data from all plots, 2010–2023).

### 3.6. Microhabitat Use

**Figure 7:**
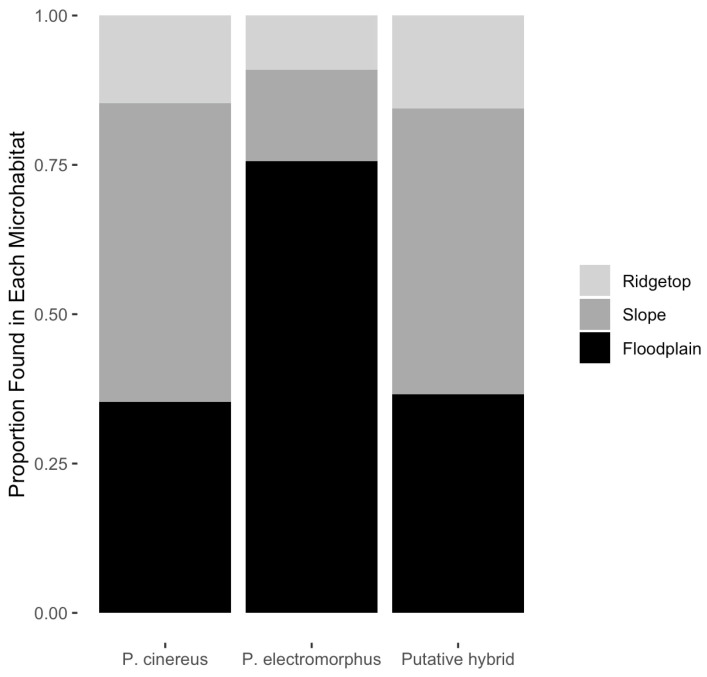
Mosaic plot of the relative frequency of salamander occurrence in the three sampled forest microhabitats (floodplain, slope and ridgetop, 2010–2023) at Wooster Memorial Park for *P. cinereus* (n = 808), *P. electromorphus* (n = 221) and their probable hybrids (n = 186). A X^2^ test for independence found that microhabitat preference was significantly different between *P. cinereus* and *P. electromorphus* (X^2^ = 117.1, df = 2, *p* < 0.001).

Of 808 *P. cinereus* detected for which microhabitat was recorded, 240 (35.3%) were found in the floodplain microhabitat, 404 (50.0%) were found in the slope microhabitat and 80 (14.7%) were found in the ridgetop microhabitat (Table 5, Figure 7). A X^2^ goodness of fit test indicated that the ridgetop microhabitat was used significantly less often and the slope microhabitat was used significantly more often than expected by chance alone (X^2^ = 152.2, df = 2, *p* < 0.001).

Of 221 *P. electromorphus* detected for which microhabitat was recorded, 167 (75.6%) were found in the floodplain microhabitat, 34 (15.4%) were found in the slope microhabitat, and 13 (9.0%) were found in the ridgetop microhabitat (Table 5, Figure 7). A X^2^ goodness of fit test indicated that both the ridgetop and slope microhabitats were used significantly less often and the floodplain microhabitat was used significantly more frequently than expected by chance alone (X^2^ = 178.7, df = 2, *p* < 0.001). Further, a X^2^ test for independence found that microhabitat preference was significantly different between these two species (X^2^ = 117.1, df = 2, *p* < 0.001).

Of 186 probable hybrids detected for which microhabitat was recorded, 68 (36.6%) were found in the floodplain microhabitat, 89 (47.8%) were found in the slope microhabitat, and 29 (15.6%) were found in the ridgetop microhabitat (Table 5, Figure 7). A X^2^ goodness of fit test indicated that the ridgetop microhabitat was used significantly less often than expected by chance alone (X^2^ = 29.9, df = 2, *p* < 0.001) but that floodplain and slope microhabitats were used at similar frequencies (X^2^ = 2.8, df = 1, *p* = 0.094).

### 3.7. Cover Object Use

**Figure 8:**
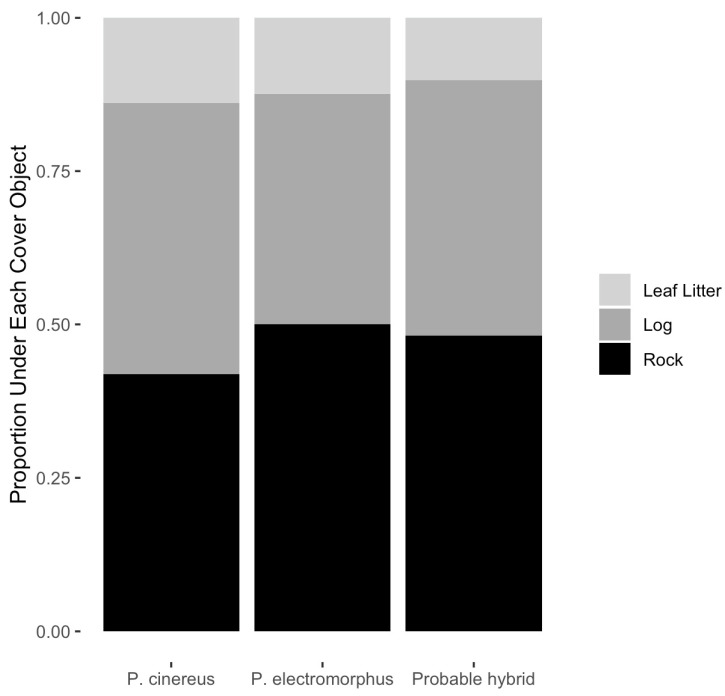
Mosaic plot of the relative frequency of salamander occurrence among cover object types (2010–2023) at Wooster Memorial Park for *P. cinereus* (n = 823), *P. electromorphus* (n = 282) and their probable hybrids (n = 245). While there was a trend for *P. electromorphus* to use more rock cover objects than *P. cinereus*, a X^2^ test for independence found that cover object preferences were not significantly different between these two species (X^2^ = 4.6, df = 2, *p* = 0.06).

Of 823 *P. cinereus* detected for which cover object type was recorded, 345 (41.9%) were found under rocks, 364 (44.2%) were found under logs and 114 (13.9%) were found in the leaf litter (Table 6, Figure 8). A X^2^ goodness of fit test indicated that leaf litter was used significantly less frequently (X^2^ = 141.2, df = 2, *p* < 0.001), but there was no significant difference in cover object preference between rocks and logs (X^2^ = 0.5, df = 1, *p* = 0.48).

Of the 282 *P. electromorphus* detected, for which cover object type was recorded, 141 (50.0%) were found under rocks, 106 (37.6%) were found under logs and 35 (12.4%) were found in the leaf litter (Table 6, Figure 8). A X^2^ goodness of fit test indicated that leaf litter was used significantly less frequently (X^2^ = 62.1, df = 2, *p* < 0.001) and rocks were preferred to logs (X^2^ = 5.0, df = 1, *p* = 0.026). However, a X^2^ test for independence found that cover object preferences were not significantly different between these two species (X^2^ = 4.6, df = 2, *p* = 0.06).

Of 245 probable hybrids detected for which cover object type was recorded, 118 (48.2%) were found under rocks, 102 (41.6%) were found under logs and 25 (10.2%) were found in leaf litter (Table 6, Figure 8). A X^2^ goodness of fit test indicated that leaf litter was used significantly less frequently (X^2^ = 60.5, df = 2, *p* < 0.001), but there was no significant difference in usage between rocks and logs (X^2^ = 1.2, df = 1, *p* = 0.286).

### 3.8. Environmental Conditions

**Figure 9:**
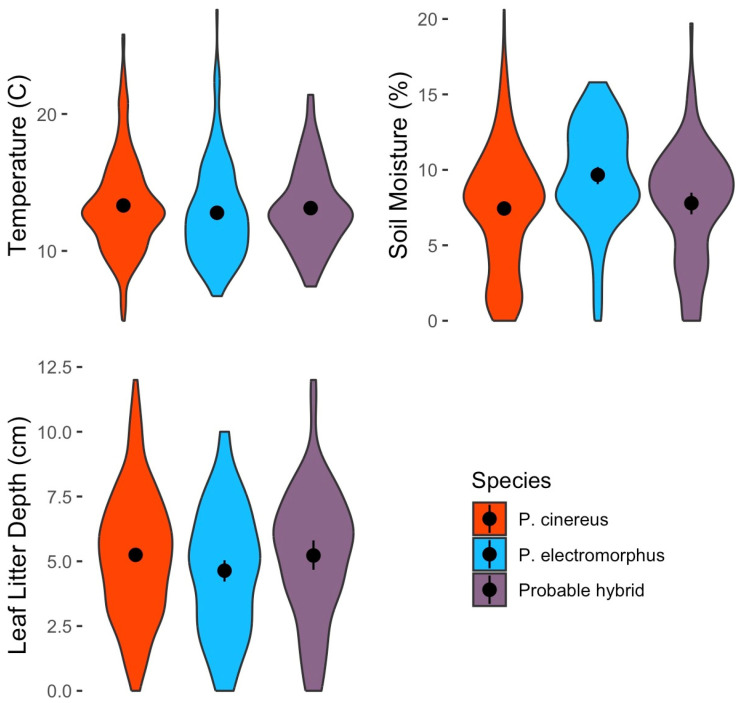
Violin plot comparing soil temperature (°C), soil moisture (%) and leaf litter depth (cm) at the capture point for *P. cinereus* (n = 416), *P. electromorphus* (n = 114) and their probable hybrids (n = 62). Means ± 95% C.I. are shown. There were no significant differences in soil surface temperature or leaf litter depth, but soil moisture was significantly greater for *P. electromorphus* (F = 8.53, *p* < 0.001).

Soil temperature, soil moisture, and leaf litter depth data from the point of capture were available from 416 *P. cinereus*, 114 *P. electromorphus* and 62 probable hybrids. A multivariate GLM indicated significant differences among groups (Wilks’ Lambda = 0.965, F = 3.47, *p* = 0.002). Subsequent univariate tests showed no significant differences among species in soil temperature (F = 0.71, *p* = 0.494) or leaf litter depth (F = 1.88, *p* = 0.153; Figure 9). However, there were significant differences in soil moisture levels among species (F = 8.53, *p* < 0.001; Figure 9). Specifically, *P. electromorphus* was found at significantly greater soil moisture compared to both *P. cinereus* (Tukey post hoc test: *p* < 0.001) and probable hybrids (*p* = Tukey post hoc test: *p* = 0.010). Soil moisture was not significantly different between *P. cinereus* and probable hybrids (Tukey post hoc test: *p* = 0.966).

### 3.9. Adult Sex Ratios and Reproductive Activity

Of 327 adult *P. cinereus* detected for which a sex could be confidently assigned, 245 were identified as male (74.9%) and 82 were identified as female (25.1%). Of the females, ten were gravid (12.2%). Of the ten gravid females, six were found in April and four were found in October. Of 87 adult *P. electromorphus* detected in this study for which a sex could be confidently assigned, 33 were identified as male (37.9%) and 54 were identified as female (62.1%). Of the females, five were gravid (9.3%, four in April, one in November). Of 72 adult probable hybrids found in this study for which a sex could be confidently assigned, 44 were identified as male (61.1%) and 28 were identified as female (38.9%). Of the females, three were gravid (10.7%, one in April and two in October). As expected, eggs were never found during plot searches, but the distribution of hatchlings among microhabitats showed patterns that mirrored overall habitat preferences for each species (Table 7, sample size too small for statistical analysis).

### 3.10. Body Size and Condition

**Figure 10:**
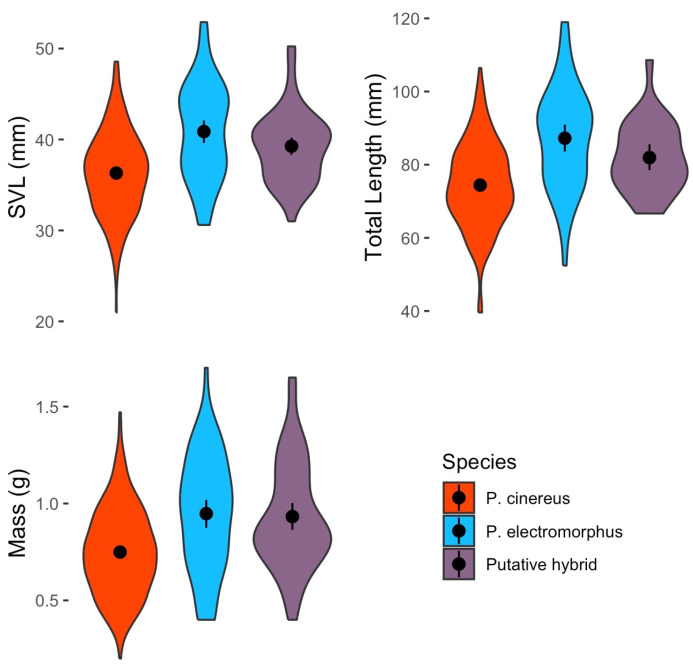
Violin plot comparing adult snout–vent length (SVL, mm), total length (TL, mm) and live body mass (g) for *P. cinereus* (n = 416), *P. electromorphus* (n = 108) and their probable hybrids (n = 62). Means ± 95% C.I. are shown. Individuals of *P. cinereus* were significantly smaller and of significantly lower body mass than individuals of *P. electromorphus* and probable hybrids (SVL: H = 41.2, df = 2, *p* < 0.001; TL: H = 40.3, df = 2, *p* < 0.001; live body mass: H = 33.1, df = 2, *p* < 0.001).

**Figure 11:**
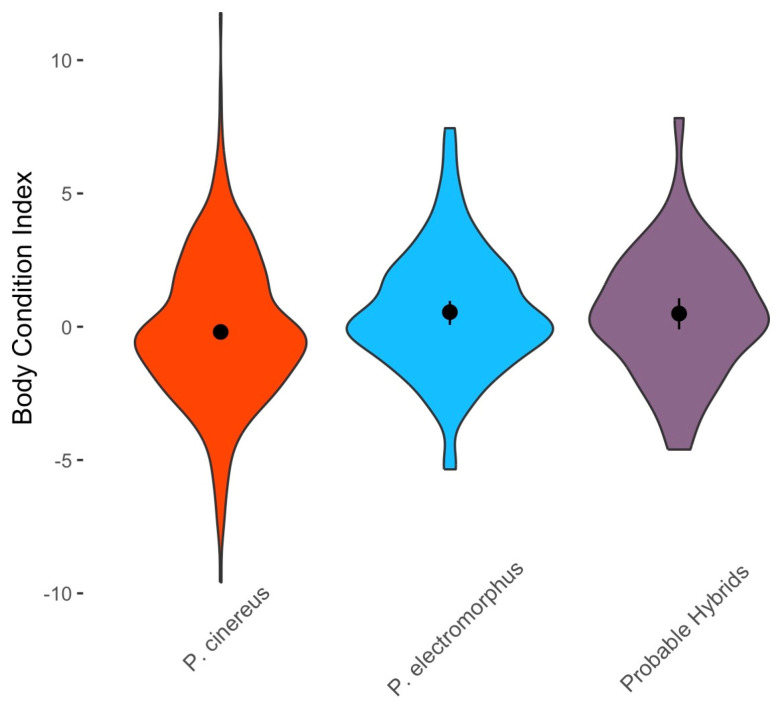
Violin plot comparing body condition index for *P. cinereus* (n = 289), *P. electromorphus* (n = 63) and their probable hybrids (n = 52). Body condition index represents the residuals from a linear regression of body mass and SVL. Means ± 95% C.I. are shown. No significant differences in body condition were found among *P. cinereus*, *P. electromorphus* and probable hybrids (F = 2.76, df = 2, *p* = 0.065).

Using only data from adults, there were statistically significant differences among groups (SVL: H = 41.2, df = 2, *p* < 0.001; TL: H = 40.3, df = 2, *p* < 0.001; live body mass: H = 33.1, df = 2, *p* < 0.001; Figure 10). Post hoc tests revealed that *P. cinereus* adults were significantly smaller (in both SVL and TL) and had significantly lower body mass than both *P. electromorphus* adults and probable hybrid adults (all *p* < 0.001 and significant after Bonferroni correction; Figure 10). However, *P. electromorphus* adults and probable hybrid adults did not significantly differ from one another (all *p* > 0.07). An analysis of body condition showed no significant differences among *P. cinereus*, *P. electromorphus* and probable hybrids (F = 2.76, df = 2, *p* = 0.065; Figure 11). Mean body length and body mass estimates for all life stages are presented in Table 8.

Mann–Whitney tests comparing field-measured body mass, SVL and TL did not find any significant sexual dimorphism between adult male and female *P. cinereus* (all *p* > 0.2). A similar analysis also showed no significant differences among adult male and female *P. electromorphus* in these traits (all *p* > 0.1; Table 8).

**Figure 12:**
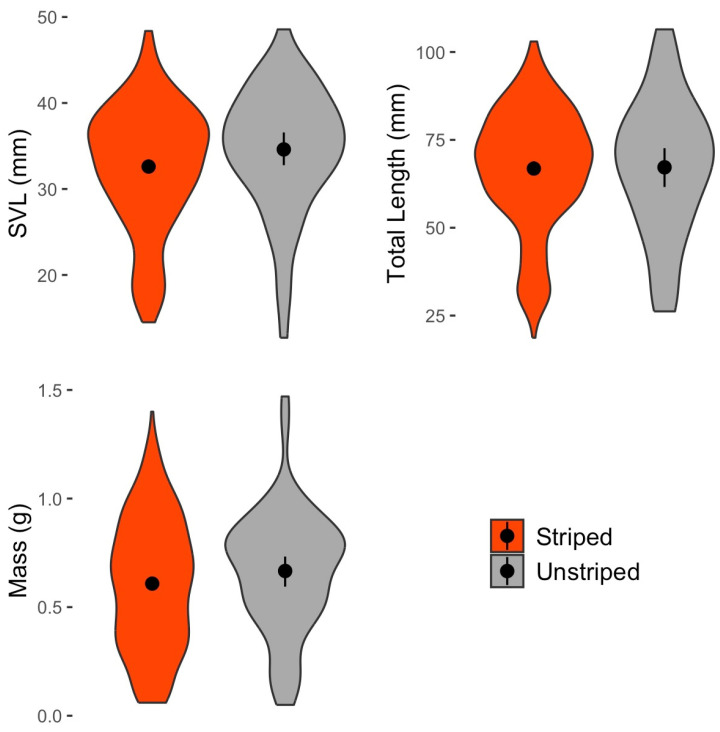
Violin plot comparing snout–vent length (SVL, mm), total length (TL, mm) and body mass (g) for the striped (n = 342) and unstriped (n = 59) color morphs of *P. cinereus*. Means ± 95% C.I. are shown. There were no statistically significant differences in SVL (*p* = 0.043, not significant after Bonferroni correction), TL (*p* = 0.996) or live body mass (*p* = 0.160).

Examining the striped and unstriped morphs of *P. cinereus*, we also did not find statistically significant differences in SVL (U = 8427, *p* = 0.043, not significant after Bonferroni correction), TL (U = 6253, *p* = 0.996) or body mass (U = 8648, *p* = 0.160; Figure 12). An analysis of body condition by using the unstandardized residuals from a SVL–body mass linear regression also showed no significant differences in body condition among color morphs (U = 8736, *p* = 0.632).

### 3.11. Estimates of the Frequency of Tail Damage

Of 551 *P. cinereus* for which the presence of tail damage was recorded, 22 had evidence of tail damage (4.0%). Of these, seven were the unstriped morph (31.8%) and 15 were the striped morph (68.2%). Of 139 *P. electromorphus*, five had evidence of tail damage (3.6%). Of 104 probable hybrids, one had evidence of tail damage (1.0%).

Environmental differences between color morphs of *P. cinereus*.

**Figure 13:**
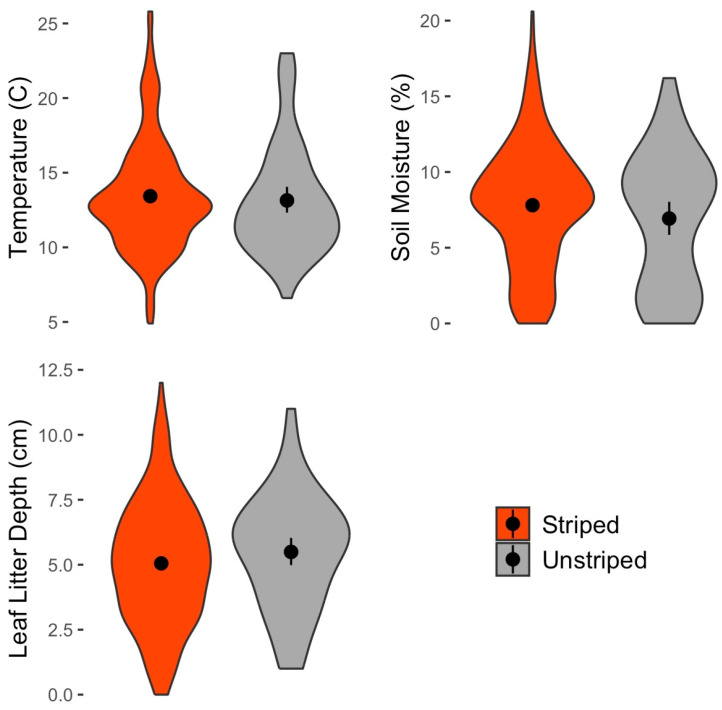
Violin plot comparing soil surface temperature (°C), soil moisture (%) and leaf litter depth (cm) at the capture point for the striped (n = 410) and unstriped (n = 70) color morphs of *P. cinereus*. Means ± 95% C.I. are shown. Soil temperature (U = 11,594, n = 447, *p* = 0.312), soil moisture (U = 9593, n = 415, *p* = 0.287) and leaf litter depth (U = 9143, n = 403, *p* = 0.121) were all not significantly different between the color morphs.

Of the 484 *P. cinereus* for which color morph was recorded, 413 were the striped color morph (85.3%), and 71 (14.7%) were the unstriped color morph. The frequency of the two color morphs was relatively consistent over the years, showing no obvious long-term change (Table 9). Soil temperature (U = 11,594, n = 447, *p* = 0.312), soil moisture (U = 9593, n = 415, *p* = 0.287) and leaf litter depth (U = 9143, n = 403, *p* = 0.121) at the capture point were all not significantly different between the two color morphs (Figure 13).

The distribution of striped individuals differed strongly among microhabitat types (X^2^ = 123.7, df = 2, *p* < 0.001), with more than expected found in slope microhabitats and fewer than expected found in floodplain and ridgetop microhabitats. The distribution of unstriped individuals among microhabitat types followed a similar pattern (X^2^ = 35.7, df = 2, *p* < 0.001). These microhabitat preferences, however, did not significantly differ by color morph (X^2^ = 4.59, df = 2, *p* = 0.101).

Similarly, the distribution of striped individuals differed strongly among cover object types (X^2^ = 103.1, df = 2, *p* < 0.001), with more than expected found under rocks and fewer than expected found under logs and leaf litter. The distribution of unstriped individuals among cover object types followed a similar pattern (X^2^ = 9.33, df = 2, *p* = 0.009). These cover object preferences, however, did not significantly differ by color morph (X^2^ = 2.63, df = 2, *p* = 0.268).

### 3.12. Estimated Frequency of Hybrids

Including all plot-based data from all years, 1533 small *Plethodon* salamanders were sampled, and 245 of these were field identified as probable *P. cinereus* × *P. electromorphus* hybrids (16.0%). Considering only the long-term monitoring plots, 49 probable hybrids were identified out of the 506 small *Plethodon* salamanders captured in total (9.7%).

### 3.13. Mated Pairs

Twenty-nine naturally occurring male–female mated pairs were found under cover objects over the course of the study. Ten of these (34%) were *cinereus–cinereus* pairs and two (7%) were *electromorphus–electromorphus* pairs. Five (17%) were *cinereus–electromorphus* pairs, and the remaining twelve pairs involved probable hybrids in various forms (41%; Table 10).

### 3.14. Literature Review of Long-Term Field Studies of Plethodontid Salamanders

Including the present study, we found 22 published papers that met our search criteria, some on a single species, some on multiple species. These studies reported information on a total of 21 plethodontid species in seven genera using various methods on diverse topics (Table 11). The earliest study began in 1947 (on *E. eschscholtzi* by Stebbins [81]), and the longest duration study we found was 29 years (on *Speleomantes strinatii* by Salvidio et al. [82]). The average study duration was 12.8 years (±6.5 SD; median: 13 years). Geographically, most studies were conducted in the USA (in eight different states, primarily in the east), with two studies in Canada and two in Italy (Table 11). The most commonly studied species was *P. cinereus* (six studies), followed by the *P. jordani* complex (four studies), the *D. ochrophaeus* complex (three studies), *Desmognathus quadramaculatus* (two studies) and *Speleomantes strinatii* (two studies), with all other species being represented by a single study (or a series of related publications based on the same data).

## 4. Discussion

In our study, we detected a total of nine salamander species (seven plethodontids) in the permanent forest plots at WMP, plus one hybrid form, five frog and two snake species. As expected, the most common salamander species were detected quickly, but more uncommon species were often not detected for several years. Despite repeated sampling, several rare species (e.g., *H. scutatum*; Table 3) were not detected until near the end of the ten-year period (Figure 3). This underscores the challenge associated with comprehensive biodiversity inventories, as it might take dozens or even hundreds of sampling events to sample rare or difficult-to-detect species [100,101,102]. Our results emphasize that sampling effort can strongly influence perceptions of species abundance [103]. To maximize the chance of detecting declines and to produce useful baseline information for future comparisons, we emphasize the importance of long time series and consistency in personnel and methodology.

Our baseline abundance densities were derived from area and time-constrained diurnal plot searches using a methodology and sampling effort that did not change during the ten-year sampling of the long-term monitoring plots. Thus, changes in abundance over time should be primarily influenced by changes in detection probability and actual changes in abundance (rather than changes in sampling effort or sampling protocols). Estimated densities ranged from 0.01 to 2.70 individuals per 100 m^2^, depending on the species and microhabitat sampled (Table 3). Of the four species for which sample sizes permitted statistical analysis, three showed no significant trends in abundance over time (*E. bislineata*, *P. cinereus* and *P. electromorphus*). Since there is no previous baseline abundance information available, it is impossible to say if abundances are different now compared to the past, but populations of these three species appear to be stable during the observed time interval.

Our overall pooled observed density of *P. cinereus* (average 1.6 individuals per 100 m^2^ plot or 0.016 per m^2^; Table 3) is lower than other estimates reported in the literature (ranging from 0.05 to 3.3 individuals per m^2^ [25,28,69,104]). However, our estimates were generated from daytime counts of surface-active individuals and, as such, are expected to be underestimates of the actual density. Nighttime counts tend to be substantially higher [105], as are capture–recapture estimates compared to counts of surface-active individuals [28]. In addition, hybrids are not included in our *P. cinereus* totals, and we also conducted some of our sampling during warm months (e.g., July and August). Considering only data from the spring and fall months brings the average up to 1.88 individuals per 100 m^2^ plot (0.0188 per m^2^). The maximum number of *P. cinereus* observed in a single plot was 19 (0.19 per m^2^), but even this value is on the low end of estimates from the literature. It is also conceivable that abundance estimates from coverboard arrays may present important biases compared to unmanipulated environments [106]. Grant et al. [28] also found that range-wide estimates of abundance for *P. cinereus* were at their lowest in a band from 40 to 42 degrees north latitude, which coincides with the latitude at WMP. The fact that WMP is a relatively small forest fragment (<200 ha) surrounded by agriculture and development may also negatively influence abundance patterns compared to more extensive protected forest areas [107]. These differences in methodology imply that comparisons across time within a study are likely more informative than comparisons of abundance among different studies.

The density estimates of *E. bislineata* reported here are also underestimates for many of the same reasons, but also because this species is most commonly encountered directly adjacent to streams, which we did not sample in this study. Our overall density estimate for *P. electromorphus* (0.43 individuals per 100 m^2^ or 0.0043 per m^2^; Table 3) is intermediate compared to Waldron et al. [56], who reported densities ranging from 0.06 to 1.8 individuals per 100 m^2^.

Unlike the other three species, which showed no pattern in abundance over time, *P. glutinosus* underwent a precipitous decrease over the course of our study (Figure 4). While never super-abundant, in the early years, finding one or several *P. glutinosus* in a plot search was a common occurrence. For example, in the first three years of the study (2014–2016), we found a total of 30 *P. glutinosus* in the long-term monitoring plots in 62 plot searches. By contrast, in the last three years of the study (2021–2023), we found a total of two individuals in 70 plot searches. Since there were no significant temporal trends in soil temperature, soil moisture or leaf litter depth (see Results), *P. glutinosus* declines at WMP are difficult to attribute to changing detectability over time. Highton [73] reported abundance information for *P. glutinosus* from 30 sites in 13 U.S. states sampled between the 1960s and the 1980s and compared these earlier numbers to revisits of the same sites in the 1990s. Of the 30 sites sampled, *P. glutinosus* abundance was lower in the 1990s in 28 cases (87%) and was greater in only 2 (13%). Using Highton’s collections and field notes, other researchers resampled sites in Great Smoky Mountains National Park in 2009 and also found statistically significant declines in abundance for *P. glutinosus* [108]. Thus, other studies on this species have found similar decreases in abundance over time. However, the cause(s) of this apparent decline have not been identified. The habitat at WMP remains protected and in good condition, and there has been no reported detection of Bd in 46 individual *P. glutinosus* in Ohio (including 25 individuals sampled at WMP; [109]). Invasive earthworm abundance was not statistically associated with *P. glutinosus* abundance, either (Table 4). Climate change impacts are another possibility (see [110]), but clearly, more investigation is needed to assess the scope and mechanisms of *P. glutinosus* decline.

### 4.1. Factors Influencing Surface Activity and Abundance

O’Donnell et al. [111] found that time since rain was one of the most important factors influencing surface activity in *P. serratus*. Surprisingly, only *E. bislineata* had a similar negative association with time since rain in our study; the other species had no significant relationship with this variable (Table 4). This could be related to a much higher proportion of individuals being found in the leaf litter in the O’Donnell et al. [111] dataset (72% in *P. serratus*) compared to our data (13.9% in *P. cinereus* and 12.4% in *P. electromorphus* (Table 6; Figure 8). Cover objects like rocks and logs are important refuges of the cooler, wetter microclimates needed by plethodontid salamanders. In contrast, the leaf litter dries rapidly [112]. Since a large majority of our detections of most species were not in the leaf litter but under cover objects (Table 6), this may reduce the negative effect that lack of rainfall might otherwise have.

The heterogeneous environmental conditions experienced by free-living organisms often have a profound influence on their physiological state and can ultimately affect their behavior, survival and reproductive success [113]. Yet, these heterogeneous environmental conditions, such as temperature and moisture, are often fine-scale and subtle [77]. The plots in this study were placed strategically in areas of different topography that reflect some of this environmental heterogeneity, especially related to moisture. Specifically, we sampled drier ridgetop forest high in the landscape, wetter, low-lying floodplain forest and the slopes in between them. While all four species examined occurred in all forest microhabitats, the type of forest microhabitat nonetheless had a very strong effect on abundance in all four species. All species had their lowest abundance in the ridgetop forests, which were also the driest microhabitats we sampled (Table 3). Most species showed their maximum abundance in the slope microhabitat, where perhaps it tends to be neither too dry nor too persistently wet (Table 3). The one exception to this pattern was in *P. electromorphus*, which was most abundant in floodplain forests and least abundant in the slope microhabitat.

### 4.2. Ecological Differences Between P. cinereus and P. electromorphus

Interspecific competition has often been demonstrated to have a powerful outcome on evolutionary trajectories when competitors occur in sympatry [52,114]. Though it has been hypothesized that asymmetric competition might cause the decline and eventual extinction of *P. electromorphus* in areas of sympatry with *P. cinereus* [49], this hypothesis is not supported by our long-term data at WMP (Figure 4). Another way to explain the ongoing coexistence of these species in sympatry is to hypothesize that divergence in sympatry has prevented competitive exclusion [114]. If divergence in sympatry has been favored by selection for competition avoidance, there should be strong differences between allopatric and sympatric populations of the same species. Deitloff et al. [54] examined allopatric and sympatric populations of *P. cinereus* and *P. electromorphus* and found no consistent differences in diet, cover object use or cranial morphology. However, our results suggest a substantial degree of habitat isolation in sympatry between these two species at WMP. Specifically, we found a strong tendency for *P. electromorphus* to occur in floodplain environments and to avoid slope microhabitats where *P. cinereus* is most common (Table 5, Figure 7). Similarly, we found *P. electromorphus* to occur in significantly moister soil than *P. cinereus* (Figure 9). These clear differences in sympatric habitat use documented between *P. cinereus* and *P. electromorphus* at WMP may result from competitive interactions and thus be a result of niche differentiation (i.e., ecological character displacement; [114]). However, further comparative studies of microhabitat use of these species in allopatry are needed to more fully test this possibility [45].

Our observations on microhabitat differences between *P. cinereus* and *P. electromorphus* are consistent with Waldron et al. [56], who also found *P. electromorphus* in wetter conditions, but are inconsistent with the results reported by Hedeen [55], who found *P. electromorphus* restricted to drier, ridgetop environments compared to *P. cinereus*. Other authors describe *P. electromorphus* habitat as primarily wooded slopes and “rarely occurring on dry hilltops or the valley floors” (see summary in [115]). Our data on *P. electromorphus* habitat use at WMP markedly departs from this characterization and shows that they tend to avoid wooded slopes and are quite abundant on the valley floor (Figure 7). Why microhabitat use apparently differs so strongly throughout the geographic range of *P. electromorphus* is unclear.

We also noted a statistically significant preference for rocks over logs or leaf litter as cover objects (Table 6, Figure 8). Since *P. cinereus* had no significant preference for rocks versus logs, this may also be an axis of niche differentiation between these two species. However, other authors have stated that *P. electromorphus* has a very strong preference for rocks and “are rarely found under any other type of cover” [116]. At WMP, we detected *P. electromorphus* under rocks 50% of the time, under logs 37.6% of the time and in the leaf litter 12.4% of the time. Thus, while there is a preference for rocks, it does not appear as strong a preference as others have previously suggested.

Besides microhabitat use, soil moisture and cover object preference, we did not find any other ecological differences in the environmental conditions used by *P. cinereus* and *P. electromorphus*. However, we did find significant differences in body size between the two species, with *P. electromorphus* averaging larger and heavier than *P. cinereus* (Table 8). These two species also had notable differences in estimated sex ratios at WMP, with 75% of confidently sexed adult *P. cinereus* being male, while only 37% of confidently sexed adult *P. electromorphus* were male. This is a strong pattern and has been reported previously [115,117]. The preponderance of females in *P. electromorphus* could possibly correspond to different phenological patterns of subterranean egg brooding in these two species (which is only known to be performed by females in *P. cinereus* [118]). Female subterranean egg brooding is assumed to be the same in *P. electromorphus* [116,117], but if this is the case, it should increase the proportion of males at the surface, which is not consistent with our data. Since male parental care is unknown in plethodontids [119], there must be some other reason for the low proportion of males.

We also noted differences in the phenological patterns of *P. cinereus* and *P. electromorphus*. While both species generally had the expected peaks of activity in the spring and fall with a decrease in activity in the hotter months, *P. electromorphus* was more strongly active in the early spring (especially April; see Figure 6). In contrast, *P. cinereus* was most active in the later fall months, especially October and November (Figure 6). The appearance of recent hatchlings at the surface also showed distinct phenological differences between *P. cinereus* and *P. electromorphus*, with *P. cinereus* hatchlings being most abundant in October and November and *P. electromorphus* hatchlings being most abundant in April and May. First noted by Pfingsten [115,117] and confirmed here, this further suggests phenological differences in reproduction and egg brooding. Sampling in additional months outside of April to November might further reveal phenological differences. These differences in the timing of surface activity and reproduction could also be related to competition avoidance (temporal niche partitioning; [120]). Comparative data on phenology in allopatry and sympatry would be helpful to assess this possibility.

### 4.3. Hybridization

Reproductive isolation is usually maintained among species pairs that occur in sympatry by various isolating barriers, either operating alone or in concert [121,122]. These barriers are often classified as pre-mating barriers (e.g., habitat, behavioral or phenological differences [123]) or post-mating barriers (e.g., gametic incompatibility, hybrid inviability or hybrid sterility [124]). Our data suggest that pre-mating barriers exist in sympatry at WMP between *P. cinereus* and *P. electromorphus*, but that post-mating barriers likely do not. Specifically, habitat isolation may be an important pre-mating barrier that reduces encounter rates between these species during the breeding season (Table 5 and Figure 7). Differences in cover object use and reproductive phenology may also partly contribute to reproductive isolation (Figure 6).

However, these isolating barriers are apparently “leaky” as DNA sequence data reported in [61] estimated that between 8 and 33% of the 36 individuals genotyped from WMP were *P. cinereus* × *P. electromorphus* hybrids, depending on the analysis used. Highton [50] estimated a frequency of hybridization at a nearby site at 10 out of approximately 150 individuals (6.7%). Our estimates of the frequency of *P. cinereus* × *P. electromorphus* hybrids from morphological characterization of individuals in the field ranged from 9.7% (long-term monitoring plot data only) to 16.0% (all plot data) and thus are within the range of those reported in [50,61]. While we reiterate that individuals field-identified as probable hybrids were not genotyped, the surprisingly high frequency of putative interspecific and hybrid-parental mated pairs (59%) compared to pure parental (41%) mated pairs found in the field underscores the apparent high frequency of hybridization at WMP (Table 10). Mallet [125] estimated that on a per-individual basis, the frequency of natural gene flow between species that hybridize in sympatry is between 1 in 100 and 1 in 10,000. Even if the true amount of gene flow between *P. cinereus* and *P. electromorphus* is on the lower end of the estimates from [61], this still seems to be an unusually large amount of interspecific gene flow that is likely influencing fitness outcomes.

Probable hybrids appear to be in many ways ecologically similar to or intermediate between the parental species. For example, cover object preferences were similar to the parental species (Figure 8), but microhabitat preference was more similar to *P. cinereus* than *P. electromorphus* (Figure 7). On the other hand, probable hybrids tended to be larger, heavier (Table 8; Figure 9) and in better body condition (Figure 11). Differential habitat use between *P. cinereus* and *P. electromorphus* was discussed above in the context of ecological character displacement and competition avoidance, but these differences could also be interpreted as hybridization avoidance. This interpretation assumes, however, that hybrids are of lower fitness than offspring from pure parental matings, which has yet to be demonstrated. While body condition indices are relatively crude measures of physiological condition [126], our body condition data did not suggest that hybrids are in poorer body condition than pure parental individuals. In fact, probable hybrids had the highest overall mean body condition index, even though the differences were non-significant (Figure 11). Additional study is needed to ascertain the evolutionary consequences of gene flow between these species and to confirm these data based on field identifications. Estimates of the growth, survival and reproductive success of marked and genotyped individuals in the field would be particularly valuable and would be an important test of the patterns reported here based on morphological characterization in the field.

### 4.4. Color Morph Results

Our data showing higher tail damage rates in the unstriped morph is consistent with the findings of previous studies [35,37]. At WMP, only ~15% of all *P. cinereus* are the unstriped morph, yet they represented ~32% of all instances of tail damage noted. This suggests higher predation pressure on unstriped morphs. Other studies have suggested that the unstriped morph is adapted to warmer and drier climate conditions compared to the striped morph, which is often found in cooler, wetter conditions [31,32,33,34]. However, our results do not show any significant differences in soil temperature, soil moisture or leaf litter depth between the color morphs (Figure 13). These results are consistent with Anthony et al. [40], who also found no differences in temperature among color morphs, but inconsistent with Petruzzi et al. [127], who did find temperature differences. As suggested by these authors, the relationship between color morph and temperature patterns may be more complex than is often presumed and may vary among sites according to variables that we did not measure. We also found no differences among color morphs in microhabitat or cover object preferences. Color morph frequency did not vary notably over time at WMP, with the 95% confidence intervals overlapping for all years (2014–2023; Table 9). This suggests no obvious signature of the influence of climate warming on color morph frequencies, as has previously been debated in other studies [31,33].

### 4.5. Body Size and Sexual Dimorphism

We found no evidence of sexual dimorphism in body mass or body length in *P. cinereus* (Table 8, Figure 10). This is consistent with some studies (e.g., [128]) but in contrast with others that have found females to be slightly larger (see summary in [69]). This difference could be driven by sampling biases, measurement or sample size differences among studies. Recently, Hantak et al. [129] reported that sexual size dimorphism in *P. cinereus* varies geographically, with some populations showing significant size differences among sexes and other populations showing no significant differences. Or perhaps the earlier maturity of males (as early as 2 years) compared to females (usually at 3 years or later; [69]), just creates a statistical impression of a difference where none actually exists. Age-standardized body size comparisons would be useful to assess this possibility. Nonetheless, there is a trend in most plethodontid species for females to be slightly larger [130].

Pfingsten [117] presented data on body size differences between male and female *P. electromorphus* from museum specimens but did not statistically analyze the data. Nonetheless, these data showed minimal differences among sexes (mean SVL in males = 47.3 mm, mean SVL in females = 48.2 mm) and are consistent with our results from field-measured animals showing no or minimal sexual dimorphism (Table 8). However, our overall means in both sexes were substantially smaller than those reported in [117]. Specifically, our average SVL for male *P. electromorphus* was 12.5% smaller than that reported by [117], and our average SVL for females was 18.2% smaller. The reasons for these body size differences are unclear and are probably not wholly attributable to different measurement methods. One possibility is that smaller average body sizes are attained at WMP because of interspecific competition with *P. cinereus*.

### 4.6. Invasive Earthworms

A variety of studies have examined the interactions between invasive earthworms and plethodontid salamanders, with conflicting results. A variety of evidence from both the field and the lab suggests that *P. cinereus* avoids interactions with some invasive earthworms and that these earthworms may degrade forest floor habitat for salamanders [66,67,131,132]. On the other hand, several other studies have provided evidence that invasive earthworms can benefit plethodontid salamanders by improving diet quality and by creating a beneficial underground habitat with their burrowing behavior [133,134,135]. Earthworm burrows have been shown to reduce predation and increase over-winter survival in *P. cinereus* [133]. Results from the current study are more consistent with the negative impacts of invasive earthworms in that we found that earthworm abundance was significantly negatively associated with the abundance of *P. electromorphus* (*p* = 0.014). However, Ransom [131] also found a negative association in the field but suggested that higher earthworm abundance may allow more underground salamander activity, rather than actually decreasing salamander numbers. Thus, evidence for negative impacts from invasive earthworms on plethodontid salamanders is partly supported by our data, but nonetheless, there are complexities and alternative interpretations that need further investigation.

### 4.7. Literature Review

According to [24], there are currently 519 species in 28 genera in the family Plethodontidae. Therefore, only 4% of all plethodontid salamander species (21 out of 519) and only 25% of genera (7 out of 28) have been the subject of at least one long-term field study (Table 11). All of these studies have examined temperate plethodontid species. We found no long-term field studies from the Neotropics, despite the fact that Neotropical plethodontids make up approximately two-thirds of the family [136]. Further, six (29%) of the long-term field studies we found were on a single species (*P. cinereus*). While these field studies on *P. cinereus* are useful and important, data from more poorly studied species are also needed. While many excellent field studies shorter than five years’ duration have also been conducted on plethodontid salamanders, this does, nonetheless, reinforce the fact that few species have been intensively studied in the field, and a large amount of work is yet to be performed on many important topics. While we have likely missed a few relevant studies, our literature review suggests there are still many opportunities to add to our knowledge of these fascinating and important creatures.

## 5. Conclusions

Our ten-year dataset from Wooster Memorial Park revealed substantial declines in one species that was formerly fairly common (*Plethodon glutinosus*) and three other species that showed no significant trend in abundance over time. Analyses of surface activity indicated roles for soil temperature, soil moisture and time of year, but the importance of these variables varied among species. Comparisons of differences between the congeners *P. cinereus* and *P. electromorphus* showed substantial ecological overlap in sympatry, with the exception of microhabitat type, which showed marked differences between these species. Our results also support earlier work that suggested an unusually high rate of hybridization between *P. cinereus* and *P. electromorphus* at this site. These results reinforce the value of long-term ecological studies to help understand current patterns and to establish baseline information for future comparisons. Based on our review, long-term field studies of plethodontid salamanders have been rare, despite the ecological importance of these animals.

## Figures and Tables

**Table 1:** Latitude and longitude coordinates of the center of each long-term monitoring plot at WMP.

Plot	Latitude	Longitude
Floodplain 1	40°49′06.467″ N	82°01′48.366″ W
Floodplain 2	40°48′57.661″ N	82°01′25.737″ W
Floodplain 3	40°48′56.980″ N	82°01′12.327″ W
Floodplain 4	40°49′02.521″ N	82°01′45.310″ W
Ridgetop 1	40°48′46.439″ N	82°01′25.672″ W
Ridgetop 2	40°49′00.984″ N	82°01′58.350″ W
Ridgetop 3	40°48′49.892″ N	82°01′37.826″ W
Ridgetop 4	40°49′07.089″ N	82°01′57.093″ W
Slope 1	40°48′45.644″ N	82°01′21.222″ W
Slope 2	40°48′52.992″ N	82°01′25.834″ W
Slope 3	40°48′56.671″ N	82°01′07.050″ W
Slope 4	40°48′48.995″ N	82°01′43.470″ W

**Table 2:** List of species of amphibians and reptiles encountered during fieldwork at Wooster Memorial Park and their pooled detections in long-term monitoring plots (2014–2023). 0 * indicates species detected outside of formal plot searches.

*Plethodon cinereus*	358
*Plethodon electromorphus*	99
probable *P. cinereus* × *P. electromorphus* hybrid	49
*Plethodon glutinosus*	62
*Eurycea bislineata*	95
*Eurycea longicauda*	6
*Desmognathus fuscus*	0 *
*Notopthalmus viridescens*	9
*Hemidactylium scutatum*	1
*Ambystoma texanum*	0 *
*Anaxyrus americanus*	27
*Pseudacris crucifer*	6
*Lithobates sylvaticus*	16
*Lithobates clamitans*	0 *
*Lithobates palustris*	0 *
*Diadophis punctatus*	9
*Pantherophis spiloides*	2
*Lampropeltis triangulum*	0 *

**Table 3:** Mean abundances (± 1 SE) for each salamander species detected in the long-term monitoring plots (100 m^2^) at WMP (2014–2023). For *P. cinereus*, *P. electromorphus*, probable hybrids, *P. glutinosus* and *E. bislineata*, abundances were estimated from the final GLMM (see Table 4). For all microhabitats pooled and for all other species, the raw abundances are reported.

Species	Floodplain Microhabitat	Slope Microhabitat	Ridgetop Microhabitat	All Microhabitats
*P. cinereus*	1.54 (0.12)	2.70 (0.19)	0.23 (0.02)	1.6 (0.17)
*P. electromorphus*	1.29 (0.19)	0.46 (0.06)	0.53 (0.04)	0.43 (0.07)
probable hybrids	0.22 (0.16)	0.36 (0.09)	0.05 (0.03)	0.21 (0.04)
*P. glutinosus*	0.19 (0.02)	0.55 (0.05)	0.07 (0.01)	0.27 (0.04)
*E. bislineata*	0.30 (0.08)	0.75 (0.12)	0.19 (0.06)	0.41 (0.05)
*E. longicauda*	0.06 (0.03)	0.01 (0.01)	-	0.03 (0.01)
*N. viridescens*	0.04 (0.02)	0.01 (0.01)	0.07 (0.03)	0.04 (0.01)
*H. scutatum*	0.01 (0.01)	-	-	0.004 (0.004)

**Table 4:** Results of generalized linear mixed models with negative binomial structure examining activity levels and abundance patterns for *P. cinereus*, *P. electromorphus*, *P. glutinosus* and *E. bislineata* at Wooster Memorial Park, 2014–2023. Fixed effects included in activity models were day of year (Julian Day), days since a soaking rain, soil temperature, soil moisture, leaf litter depth and year. We included plot identity as a random effect. The abundance models examined habitat type, earthworm abundance and year as fixed effects, and random effects included plot identity and any significant activity factors for that species. Statistically significant variables are noted in bold.

		***P. cinereus***		***P. electromorphus***		***P. glutinosus***		***E. bislineata***	
**Surface Activity Models**	**Parameter**	**Coefficient**	***p*-Value**	**Coefficient**	***p*-Value**	**Coefficient**	***p*-Value**	**Coefficient**	***p*-Value**
	day of year	**0.003**	**0.018**	−0.003	0.158	0.002	0.173	**0.005**	**0.009**
	days since rain	0.009	0.542	−0.017	0.43	0.24	0.087	**−0.06**	**0.021**
	soil temperature	**−0.106**	**<0.001**	−0.045	0.22	0.047	0.144	**0.071**	**0.041**
	soil moisture	0.051	0.276	**0.101**	**0.008**	0.002	0.952	−0.003	0.948
	leaf litter depth	0.089	0.166	0.073	0.375	−0.041	0.572	0.005	0.942
	year	varies	0.203	varies	0.793	varies	0.427	varies	0.083
**Abundance Models**	habitat type	**varies**	**<0.001**	**varies**	**<0.001**	**varies**	**0.005**	**varies**	**0.013**
	earthworms	0.009	0.579	**−0.048**	**0.014**	−0.002	0.903	−0.017	0.274
	year	**varies**	**0.049**	varies	0.108	varies	0.42	varies	0.073

**Table 5:** Pooled number of detections of *P. cinereus*, *P. electromorphus* and probable hybrids in plot searches in three forest microhabitat types at Wooster Memorial Park, 2010–2023. Includes data from both 10 × 25 m plots (2010–2015) and 10 × 10 m plots (2014–2023).

	Floodplain	Slope	Ridgetop	Total
*P. cinereus*	285	404	119	808
*P. electromorphus*	167	34	20	221
probable hybrid	68	89	29	186

**Table 6:** Pooled number of detections of *P. cinereus*, *P. electromorphus* and possible hybrids in plot searches under three cover object types at Wooster Memorial Park, 2010–2023. Includes data from both 10 × 25 m plots (2010–2015) and 10 × 10 m plots (2014–2023).

	Rock	Log	Leaf Litter	Total
*P. cinereus*	345	364	114	823
*P. electromorphus*	141	106	35	282
probable hybrid	118	102	25	245

**Table 7:** Distribution of *P. cinereus* and *P. electromorphus* hatchlings among forest microhabitat types in the long-term monitoring plots at WMP (2014–2023). Hybrids could not be assigned at very small body sizes and are not represented here.

Species	Floodplain	Slope	Ridgetop	Total
*P. cinereus*	19	18	1	41
*P. electromorphus*	11	4	2	20

**Table 8:** Mean snout–vent length (SVL), total length (TL) and body mass (g, ± 1 SE) of *P. cinereus*, *P. electromorphus* and their probable hybrids in the long-term monitoring plots at WMP (2014–2023). “Both” includes adult males and females as well as adult-sized individuals that were not assignable to sex in the field.

Species	Life Stage	Sex	N	Mean TL (mm)	Mean SVL (mm)	Mean Live Body Mass (g)
*Plethodon cinereus*	Adult	both	302	74.2 (0.8)	36.1 (0.26)	0.75 (0.01)
	Adult	female	44	74.7 (1.8)	36.5 (0.71)	0.78 (0.03)
	Adult	male	193	74.2 (0.8)	36.7 (0.33)	0.75 (0.02)
	Juvenile	-	73	51.5 (1.3)	26.5 (0.44)	0.31 (0.01)
	Hatchling	-	38	32.9 (1.3)	18.6 (0.51)	0.12 (0.01)
*Plethodon electromorphus*	Adult	both	71	87.3 (1.9)	40.2 (0.7)	0.93 (0.04)
	Adult	female	42	86.6 (2.6)	39.4 (0.9)	0.91 (0.06)
	Adult	male	29	88.0 (2.8)	41.4 (1.1)	0.95 (0.05)
	Juvenile	-	19	52.2 (2.7)	27.2 (1.0)	0.32 (0.04)
	Hatchling	-	17	35.9 (1.5)	18.9 (0.9)	0.12 (0.02)
Probable hybrids	Adult	both	60	81.9 (1.8)	39.2 (0.5)	0.92 (0.04)
	Adult	female	21	86.8 (8.7)	39.1 (1.0)	0.98 (0.08)
	Adult	male	39	81.1 (1.6)	39.2 (0.6)	0.90 (0.04)
	Juvenile	-	-	-	-	-
	Hatchling	-	-	-	-	-

**Table 9:** Frequency of the striped and unstriped color morphs of *P. cinereus* at WMP by year. 95% confidence intervals were estimated with the binomial exact calculation.

Year	Striped	Unstriped	Total	% Striped	95% C.I.
2014	36	4	40	0.900	0.763–0.972
2015	44	7	51	0.863	0.737–0.943
2016	35	7	42	0.833	0.686–0.930
2017	11	1	12	0.917	0.615–0.998
2018	17	5	22	0.773	0.546–0.922
2019	38	9	47	0.809	0.667–0.909
2020	48	7	55	0.873	0.755–0.947
2021	42	12	54	0.778	0.644–0.880
2022	81	10	91	0.890	0.807–0.946
2023	61	9	70	0.871	0.770–0.939
all years	413	71	484	0.853	0.819–0.884

**Table 10:** Field observations of mated pairs of *P. cinereus*, *P. electromorphus* and probable hybrids at Wooster Memorial Park (2014–2023).

Combination	Number of Pairs
*cinereus-cinereus*	10
*electromorphus-electromorphus*	2
*electromorphus-cinereus*	5
hybrid-hybrid	3
male *cinereus*-female hybrid	3
female *cinereus*-male hybrid	4
male *electromorphus*-female hybrid	2
Total	29

**Table 11:** List of known field-based continuous long-term population monitoring studies of plethodontid salamanders with species, locations, sampling methodologies, citations and length of time series (5-year minimum).

Study Species	Location	Length of Time Series (Years)	Sampling Methodologies	Research Focus	Citations
*Aneides hardii*	New Mexico, USA	9 (1986–1996)	visual encounter searches	logging impact assessment	[83]
*Aneides lugubris*	California, USA	5 (2006–2010)	capture-mark-recapture	growth, age at maturity, survival	[84]
*Desmognathus aeneus*	North Carolina, USA	19 (1972–1990)	visual encounter searches	temporal trend analysis	[85]
*Desmognathus fuscus* complex	North Carolina and Tennessee, USA	6 (1993–1995 and 1998–2000)	visual encounter searches	abundance estimation	[86]
*Desmognathus monticola*	North Carolina, USA	19 (1972–1990)	visual encounter searches	temporal trend analysis	[85]
*Desmognathus ochrophaeus*	North Carolina, USA	7 (1970–1976)	removal sampling, capture-mark-recapture	growth, survival and life history	[87]
*Desmognathus ochrophaeus*	North Carolina, USA	19 (1972–1990)	visual encounter searches	temporal trend analysis	[85]
*Desmognathus ochrophaeus* complex	North Carolina and Tennessee, USA	6 (1993–1995 and 1998–2000)	visual encounter searches	abundance estimation	[86]
*Desmognathus quadramaculatus*	North Carolina and Tennessee, USA	6 (1993–1995 and 1998–2000)	visual encounter searches	abundance estimation	[86]
*Desmognathus quadramaculatus*	North Carolina, USA	19 (1972–1990)	visual encounter searches	temporal trend analysis	[85]
*Desmognathus wrightii*	North Carolina and Tennessee, USA	6 (1993–1995 and 1998–2000)	visual encounter searches	abundance estimation	[86]
*Ensatina eschscholtzi*	California, USA	5 (1947–1951)	visual encounter searches	longevity, movement, phenology, growth	[81]
*Eurycea bislineata*	Ohio, USA	10 (2014–2023)	visual encounter searches	temporal trend analysis	this study
*Eurycea quadridigitata*	South Carolina, USA	16 (1979–1994)	drift fence with pitfall traps	temporal trend analysis	[13]
*Eurycea wilderae*	North Carolina and Tennessee, USA	6 (1993–1995 and 1998–2000)	visual encounter searches	abundance estimation	[86]
*Gyrinophilus porphyriticus*	New Hampshire, USA	20 (1999–2018)	visual encounter searches	variation in abundance and survival	[88,89]
*Plethodon cinereus*	18 locations across the range	7 (2013–2019)	spatial capture-recapture	density estimation	[28]
*Plethodon cinereus*	Ohio, USA	6 (2003–2008)	artificial cover boards	food web dynamics	[90]
*Plethodon cinereus*	Ohio, USA	10 (2014–2023)	visual encounter searches	temporal trend analysis	this study
*Plethodon cinereus*	Ontario, Canada	18 (1999–2016)	artificial cover boards	temporal trend analysis	[91]
*Plethodon cinereus*	Virginia, USA	14 (1966–1979)	visual encounter searches	interspecific competition	[92]
*Plethodon cinereus*	Virginia, USA	17 (2005, 2008–2023)	visual encounter searches	longevity estimation	[93]
*Plethodon electromorphus*	Ohio, USA	10 (2014–2023)	visual encounter searches	temporal trend analysis	this study
*Plethodon glutinosus*	Ohio, USA	10 (2014–2023)	visual encounter searches	temporal trend analysis	this study
*Plethodon hubrichti*	Virginia, USA	17 (2005, 2008–2023)	visual encounter searches	longevity estimation	[93]
*Plethodon jordani*	North Carolina, USA	9 (1973–1981)	removal sampling	growth, age at maturity, survival	[94]
*Plethodon jordani*	North Carolina, USA	15 (1976–1990)	visual encounter searches	temporal trend analysis	[85]
*Plethodon shenandoah*	Virginia, USA	14 (1966–1979)	visual encounter searches	interspecific competition	[92]
*Plethodon shermani*	North Carolina, USA	6 (2009–2014)	capture-mark-recapture	climate change impacts	[95]
*Plethodon shermani/P. teyahalee*	North Carolina, USA	8 (2010–2017)	capture-mark-recapture	survival and fecundity	[96]
*Plethodon shermani/P. teyahalee*	North Carolina, USA	17 (1974–1990)	visual encounter searches	hybrid zone dynamics	[62]
*Plethodon shermani/P. teyahalee*	North Carolina, USA	15 (1976–1990)	visual encounter searches	temporal trend analysis	[85]
*Speleomantes strinatii*	Italy	29 (1996–2024)	removal sampling	demography, climate change impacts	[82,97,98]
*Speleomantes strinatii*	Italy	13 (1993–2005)	removal sampling	population regulation	[99]

## Data Availability

All original data presented in this study are openly available at https://doi.org/10.6084/m9.figshare.30698186.
